# The Flortaucipir PET signature in Autosomal Dominant Alzheimer's Disease due to the A431E PSEN1 mutation

**DOI:** 10.1002/alz70856_101464

**Published:** 2025-12-25

**Authors:** John M Ringman, Bryan Rowe, Claudia Alvarado, Lillibeth Barrera, Abhay P Sagare, Jiaxin Yue, Helena C Chui, Yonggand Shi

**Affiliations:** ^1^ Department of Neurology, Keck School of Medicine at USC, Los Angeles, CA, USA; ^2^ Zilkha Neurogenetic Institute, Keck School of Medicine, University of Southern California, Los Angeles, CA, USA; ^3^ Stevens Neuroimaging and Informatics Institute, Keck School of Medicine, University of Southern California, Los Angeles, CA, USA; ^4^ Department of Neurology, Keck School of Medicine, University of Southern California, Los Angeles, CA, USA; ^5^ Department of Neurology, Stevens Neuroimaging and Informatics Institute, Keck School of Medicine, University of Southern California, Los Angeles, CA, USA

## Abstract

**Background:**

The A431E mutation in PSEN1 represents a large cohort originating as a founder effect, being most prevalent in Jalisco State in Mexico. It frequently presents with spastic paraparesis, the underlying pathogenesis of which is incompletely understood. We sought to characterize the pattern of neurofibrillary tangle deposition in this subtype of AD, potentially shedding light on the etiology of this clinical manifestation.

**Method:**

We performed comprehensive clinical evaluations and flortaucipir PET (FTP) scans on 16 persons symptomatic (defined as having CDR scores greater than 0,5) with the A431E mutation and 20 persons either with ADAD mutations not associated with spastic paraparesis (*n* = 5) or having sporadic AD (*n* = 15). Dynamic raw PET images were averaged to the first scan, averaged, and registered to a T1‐weighted MRI image. Partial volume correction was performed using PetSurfer in Freesurfer 6.0 and SUVRs calculated relative to the gray matter in the inferior cerebellum. Ordinal clinical variables and SUVRs were compared between groups using two‐sided t‐tests.

**Result:**

The A431E and comparison group were comparable with regards to mean CDR‐SOB (7.0 and 6.6), MMSE scores (15.3 vs. 16.5), and sex (56 vs. 45% men) but differed markedly by age (65 vs. 43, *p* < 001). FTP was significantly elevated in widespread area of the brain in affected carriers of the A431E mutation relative to the comparison group, with these differences being greatest (*p* < 0.001) in the para‐, pre‐, and post‐central gyri, but also in the superior temporal and superior frontal gyri bilaterally. Entorhinal FTP SUVRs were non‐significantly higher in the A431E group.

**Conclusion:**

Relative to AD cases matched by disease severity, the A431E PSEN1 mutation is associated with more severe neurofibrillary tangle pathology, particularly in peri‐Rolandic cortex, likely related in some way to the spastic paraparesis occurring with this mutation.

**Funding Sources**: P30AG0665301, R01AG06901, R01AG062007, RF1AG077578, RF1AG064584, U01AG051218

